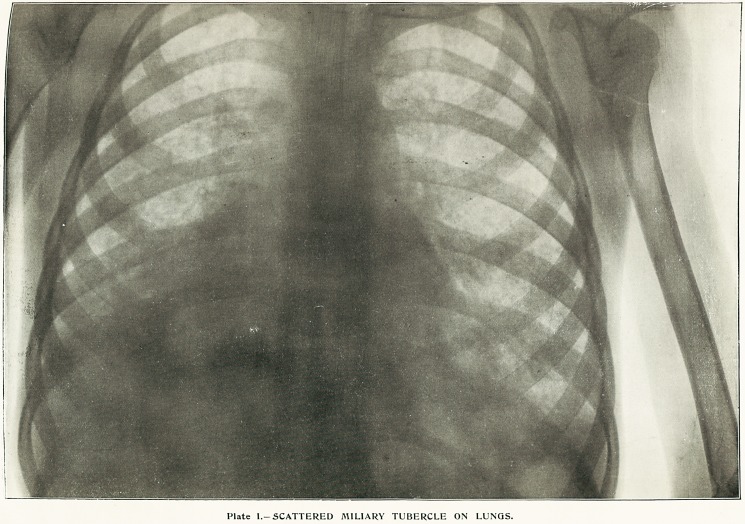# Recent Progress in Radiography

**Published:** 1899-10-07

**Authors:** David Walsh

**Affiliations:** Physician Western Skin Hospital; late Hon. Secretary, Röntgen Society, London.


### Hospital Clinics and Medical Progress.

### RECENT PROGRESS IN RADIOGRAPHY.

By David Walsh, M.D.Edin., Physician Western Skin Hospital; late Hon. Secretary, Röntgen Society, London.

To the philosophic onlooker nothing can be more remarkable than the rapidity with which new methods and principles are adopted in the world of medicine. A generation ago and the science of bacteriology was unknown; to-day it dominates the whole field of medical and surgical art. In December, 1895, Professor Röntgen announced to the Physico-Medical Society of Würzburg his since famous discovery of a new form of radiation. Four years later the " X " or Röntgen rays occupy the attention of thousands of busy workers all over the globe, and of various special learned societies and journals. They have furnished the surgeon with an additional means of exact diagnosis; they have brought relief to the wounded in war in all four quarters of the earth; they form part of the machinery of every properly equipped hospital, and they are now becoming available to the physician in dealing with purely medical diseases.

By this time it may safely be assumed that most of the readers of the present article will have a general notion of the principles of radiography. How the X-rays are generated by the passage of an electrical discharge of high tension through a glass bulb or tube, from which all but a minute fraction of air has been exhausted; how these X rays, themselves invisible, have a marvellous faculty of penetrating substances, such as wood or flesh, that are opaque to ordinary light; and how the rays are able to act upon sensitive photographic surfaces, and to cause certain chemical substances to light up or " fluoresce."

The resistance offered to the passage of the rays varies in different substances, roughly speaking, with their density. Metals are extremely opaque, and a thin sheet of lead will throw a dense shadow; whereas a thick book, like the " London Directory," causes only a light shadow. Bone resists the rays, while flesh is translucent, so that the bony skeleton can be readily examined in the living body. Fortunately, soft tissues vary as to their X-ray opacity within somewhat wide limits; blood, pus, and some of the solid organs, as the liver, offer a good deal of resistance compared with such easily penetrable tissues as cartilage and muscle. The result is that in many cases alterations of shape, position, and density of soft tissues and internal organs may be ascertained by Röntgen methods of investigation.

Shadows may be recorded in two ways—first, permanently on a photographic plate; and, secondly, as a fleeting picture on a fluorescent screen. The latter is best adapted for the examination of the chest, and it is to the consideration of that branch of practical medicine—physical diagnosis—that the present article will be mainly devoted. The possibilities of screen work have been much widened by recent improvements in apparatus. The electrolytic break, reintroduced by Wehnelt, has so greatly multiplied the power of the induction coil that the penetration of bodies of large bulk (*e.g.*, stout patients) and instantaneous Röntgen photography have both been brought within the range of practical work. The Wehnelt break interrupts with extreme frequency the current that excites the coil, and the tension of the resulting secondary or induced current is so enormously multiplied that, instead of a series of single sparks leaping across between the terminals of the spark-gap, that space is bridged over by a cable of sparks in mass as thick as one's finger. The effect of this increased energy upon the ordinary focus-tube is shown by the cracking and melting of the glass, and the volatilisation of the platinum anti-kathode. However, tubes that will withstand the intense heat generated by the new break are now on the market. Instead of the 10 or 12 volts from a secondary battery that sufficed to work the coil with the ordinary spring or mercury brake the new electrolytic appliance requires a current of 50 to 100 volts. It need hardly be remarked that great care must be taken to avoid accidents both to patients and to operator in handling apparatus of this powerful description.

With regard to physical diagnosis it is mainly in dealing with the thorax that the physician will find the new method of value. In the abdomen a record of spleen and kidneys may sometimes be obtained; the stomach can be outlined in various ways, as by the passage of a fine metal-armed bougie, and the intestines by administering bismuth or metallic mercury. Gall-stones have been occasionally recognised; cancerous masses of large size, together with dropsical and other effusions have been demonstrated, and foreign bodies of various kinds detected. The radiography of the adult abdomen, however, still presents a comparatively unbroken field to the practical worker.

In the thorax the fluorescent screen furnishes the better and quicker plan of investigation. It consists of a frame like that of a slate, over which is stretched a sheet of cardboard coated on one side with platino-cyanide of barium, one of the salts that fluoresces under the influence of the X-rays. An opaque object, as a bunch of keys, placed between the active focus-tube and the screen throws a shadow-picture upon the latter. In examining the chest the screen may be placed either in front or at the back of the patient. The examination must be made in an absolutely dark room, and the light from the focus-tube should also be excluded by wrapping the tube in black or brown paper. The eye of the observer wants some training before he can fully recognise the significance of shadows upon the screen.

The cardinal fact upon which radiography of the thorax depends is the varying resistance offered by its soft tissue contents to the passage of the rays. Healthy lung is practically translucent, blood is opaque, so too is the dense structure of the heart, of new growths, of a solid organ like the liver (limiting the lower lung), of altered density of lung substance (emphysema, phthisis), and of collections of serous or purulent fluid. From these varying physical conditions, normal and otherwise, the shadows thrown upon screen or sensitive plate afford sufficient contrast to form a readable picture.

### The Heart and Great Vessels.

It is, of course, necessary at the outset to learn the appearances of the normal heart. From the front both shape and position vary not only in relation to the stage of pulsation, but also to the movement of respiration. The latter have been given by Dr. F. H. Williams[1] as half an inch in quiet breathing, and two and a half or three inches in forced respiration, a little more on the right than on the left side. The present writer has elsewhere[2] described the appearances with the screen placed upon the anterior chest wall as follows: "From the front, then, the heart-shadow fuses on the (observer's) left with that of the sternum, beyond which the right auricle projects from the third to the fifth ribs: on the (observer's) right the auricular and ventricular line runs with a bold curve from the second to the sixth ribs. This latter line is raised and pushed upwards and outwards by the rising of the diaphragm in expiration. On the other hand, it is elongated, and carried downwards and inwards towards the sternum in forced expiration, when a light falciform line, or a broader band, defines the apex and more or less of the lower border. Ordinarily, the lower portion of the heart-shadow merges into that of the liver."

Seen from the back, with the tube midway between sternum and nipple at the level of the latter, the cardiac shadow is triangular, on the right merging into that of the spine, below into that of the liver, and on the left starting downwards and outwards from the second or third rib to the axillary line. In this left slanting border pulsation may be seen. A small portion of the heart shadow, lighter in tint than the main shadow, projects to the right of the spine. From these data enlargements of the heart can be recognised, as well as effusions into the pericardial sac. A more important point to the physician, however, is the detection of aneurism. As everyone knows, under some circumstances it is now and then extremely difficult or impossible to diagnose that condition. In the book already quoted the present writer has remarked: "Aneurisms may at times be detected by the rays in the thorax when their presence could not be demonstrated by percussion or auscultation. Several cases of unsuspected thoracic aneurism revealed by the screen have come under the observation of the present writer. In one instance he took a Röntgen photograph of the chest of an elderly asthmatic of heavy build. A good sized aneurism was seen in the left upper thorax, where, owing to the large and emphysematous lungs, it could not be diagnosed by ordinary physical signs. Indeed, an experienced physician subsequently denied in Court that an aneurism existed."[3]

### The Lungs.

An increase in the density of the lung substance deepens the tint of the resulting X-ray shadow. Thus, the consolidations of phthisis and pneumonia are recorded by a shadow deep in proportion to their density, and the same is true of œdema and congested conditions of lung. An early stage of phthisis in one apex may be detected by its slightly deeper tint when contrasted with the sound lung. At the same time the other great radiographic sign of phthisis will be visible on the screen, namely, restricted respiratory movements; instead of moving two or three inches in forced inspiration, the excursion of the diaphragm is curtailed or almost altogether absent. On the strength of these two screen observations several competent observers have succeeded in diagnosing early phthisis before the detection of tubercle bacilli in the sputum, and before the usual physical signs were recognisable. The photograph shown in Plate I. was taken at the North-Eastern Hospital for Children, London. It shows with remarkable clearness miliary tubercles scattered through both lungs. The darker tint at the base of the right lung registers adherent pleuritic membrane, a fact that the present writer confirmed post-mortem. The membrane was not greatly thickened, but was firmly adherent, and there was, of course, no effusion of fluid. These facts show that the resistance to the Röntgen rays of a normally thin and translucent membrane may be greatly increased by inflammatory processes. The observation has a distinct suggestive value in dealing with the radiography of soft tissues.

The second illustration (Plate II.) may be here briefly described. It shows the femur of an infant softened and broken as the result of acute or "scurvy" rickets. It was taken by Mr. C. G. Burton, of the North-Eastern Hospital for Children, to whose courtesy reproduction of this rare condition has been here permitted. The picture illustrates the bending and fracture of the bone, as well as the thinning of the compact wall of the femur, and the rarefaction of the cancellous tissue.

Dilated bronchi and cavities in the lung-substance may at times be demonstrated, both when empty and when full. The writer has secured a record of bronchiectatic cavities in the lung of an elderly asthmatic. Foreign bodies, as glass, bullets, or coins, can be detected in the bronchi, and in other positions inside the thorax; and a similar remark applies to solid tumours, and enlarged bronchial glands. The diagnosis between the two last-mentioned conditions and aneurism depends upon the expansile border of the vascular tumour.

The upper border of the diaphragm, or, more correctly speaking, the liver (for the muscle is radiographically translucent) affords some valuable data, besides those already referred to. In emphysema it is depressed, and limited in excursion. In enlargements of the liver and increased tension of abdominal cavity (*e.g.*, dropsy) it is higher than normal, and also limited in movement. The outline may be irregular in abscess, hydatids, sarcomata, gummata and other conditions.

Pleuritic effusions yield a dark X-ray record, especially when purulent. Their extent and progress can be accurately determined by the screen.

The present position of radiography applied to the physical diagnosis of chest conditions may be summed up as follows: In ordinary cases little more information can be gained than is available from methods previously in use. It is in exceptional cases, such as doubtful aneurism, deep patches of pneumonia, and early cases of phthisis that now and then the Röntgen rays will reveal a state of affairs discoverable by no other means in the possession of the physician. An undoubted advantage is afforded by the exact radiographic localisation of lung lesions which will doubtless be brought sooner or later within the reach of masterful modern surgery. Further the exact extent of a lung trouble can be ascertained with a degree of accuracy hitherto unattainable. Then again, the physician will find a distinct gain in esti

Plate II.—FRACTURED FEMUR IN SCURVY—RICKETS.

Plate I.—SCATTERED MILIARY TUBERCLE ON LUNGS.

mating the progress of lung and heart mischief. He may watch from day to day the clearing-up or otherwise of a pleuritic effusion, the reduction of an enlarged heart under the Schott treatment, and the increase or decrease of the consolidated areas of tubercle. The latter fact is of especial value nowadays, when the curability of early phthisis is universally recognised. By having a Röntgen photograph taken at stated intervals the physician would obtain a graphic record of the results of treatment, whereby he might be guided to a safer prognosis. The greatest enthusiast would not claim that the old methods of physical diagnosis are to be superseded by Röntgen ray investigation. It cannot be too strongly insisted upon that the rays simply furnish both physician and surgeon with a fresh weapon of accurate observation.

Where a choice is open, it is desirable that the Röntgen examination should be placed in the hands of a medical expert. Already the foundations of a flourishing unqualified practice have been laid by handing over the Röntgen examination of patients to instrument makers and electricians. So far as the physician is concerned, it is clear that any report to be of value must come from a Röntgen operator who is versed in medical matters.

[1] Brit. Med. Jour., April 16, p. 1,003. [2] "The Röntgen Rays in Medical Work." D. Walsh, M.D. Baillière and Cox, London, 1899. Second edition, p. 185. [3] Op. cit., 2nd ed., p. 188.

## Figures and Tables

**Plate II. f1:**
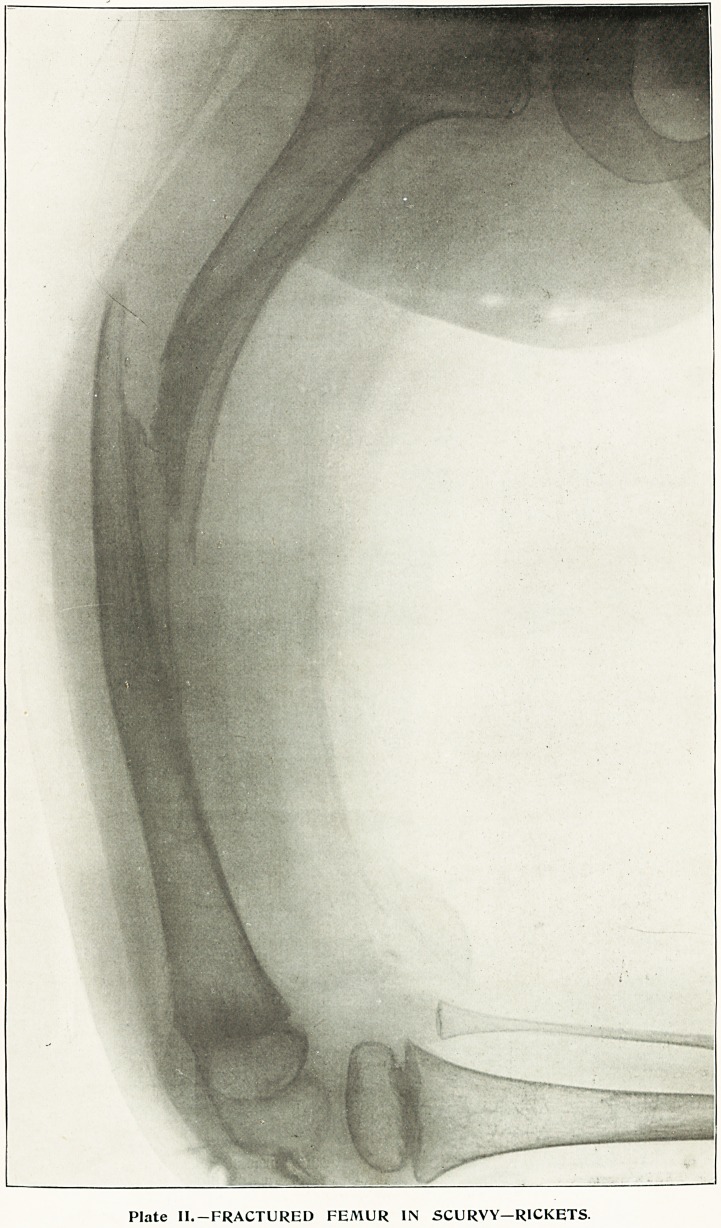


**Plate I. f2:**